# Supplementary material for: Asynchronous magnetic resonance elastography: Shear wave speed reconstruction using noise correlation of incoherent waves
Source: Magn Reson Med. 2022 Oct 27;89(3):990–1001. doi: 10.1002/mrm.29502 (PMC9792433; doi:10.1002/mrm.29502)
Supplement: Supplementary file 1 — DATA S1 MATLAB code used to generate the numerical simulations of Figure 1 [file MRM-89-990-s002.zip › k-Wave/helpfiles/example_pr_2D_fft_line_sensor.html]

2D FFT Reconstruction For A Line Sensor Example (k-Wave)


# 2D FFT Reconstruction For A Line Sensor Example

This example demonstrates the use of k-Wave for the reconstruction of a two-dimensional photoacoustic wave-field recorded over a linear array of sensor elements. The sensor data is simulated using `kspaceFirstOrder2D` and reconstructed using `kspaceLineRecon`. It builds on the Homogeneous Propagation Medium and Heterogeneous Propagation Medium examples.

- Open the file in the MATLAB Editor
- Run the file in MATLAB

## Contents

- Defining a smooth initial pressure distribution
- Defining the time array
- Simulating the sensor data
- Performing the reconstruction
- The limited view problem
- Setting the interpolation mode

## Defining a smooth initial pressure distribution

The sensor data used for the reconstruction is generated using `kspaceFirstOrder2D` in the same way as in the Initial Value Problems examples. (The reconstruction of experimental data can approached in the same fashion by substituting the numerical sensor data with experimental measurements.) It is important to note that, by default, the function `kspaceFirstOrder2D` spatially smooths the initial pressure distribution using `smooth` (see the Source Smoothing Example). To provide a valid comparison, the output of the reconstruction code `kspaceLineRecon` must be compared to the *smoothed* version of the initial pressure used in the simulation. This can be achieved by explicitly using `smooth` before passing the initial pressure to `kspaceFirstOrder2D`.

```
% smooth the initial pressure distribution and restore the magnitude
source.p0 = smooth(source.p0, true);
```

The default smoothing within `kspaceFirstOrder2D` can be modified via the optional input parameter `'Smooth'`. If given as a single Boolean value, this setting controls the smoothing of the initial pressure along with the sound speed and density distributions. To control the smoothing of these distributions individually, `'Smooth'` can also be given as a three element array controlling the smoothing of the initial pressure, the sound speed, and the density, respectively.

## Defining the time array

Although `kspaceFirstOrder2D`
can be used to automatically define the simulation length and time-step (by default `kgrid.t_array`, `kgrid.Nt`, and `kgrid.dt` are set to `'auto'` by `kWaveGrid`), this information is also required by `kspaceLineRecon` and thus in this example the time array must be explicitly created. This can be easily achieved by directly calling the `makeTime` method of the `kWaveGrid` class. This is the same function that is called internally by the first-order simulation functions when `kgrid.t_array` is set to `'auto'`.

```
% create the time array
kgrid.makeTime(medium.sound_speed);
```

## Simulating the sensor data

The FFT reconstruction function `kspaceLineRecon` requires data recorded along an equally spaced line-shaped array of sensor points. A sensor with this shape can be created by defining a binary sensor mask matrix with a line of 1's along the first matrix row (i.e., a line along x = const).

```
% define a binary line sensor
sensor.mask = zeros(Nx, Ny);
sensor.mask(1, :) = 1;
```

The initial pressure distribution (along with the binary sensor mask) and the recorded sensor data returned after running the simulation are shown below.


## Performing the reconstruction

The reconstruction is invoked by calling `kspaceLineRecon` with the recorded sensor data, as well as the properties of the acoustic medium and the sampling parameters. By default, the sensor data input must be indexed as `p(time, sensor_position)`. This means the simulated sensor data returned by `kspaceFirstOrder2D` which is indexed as `sensor_data(sensor_position, time)` must first be rotated. Alternatively, the optional input parameter `'DataOrder'` can be set to `'yt'` (the default settings is `'ty'`). By setting the optional input parameter `'Plot'` to `true`, a plot of the reconstructed initial pressure distribution is also produced.

```
% reconstruct the initial pressure
p_xy = kspaceLineRecon(sensor_data.', dy, kgrid.dt, medium.sound_speed, 'Plot', true);
```

As the reconstruction runs, the size of the recorded data and the time to compute the reconstruction are both printed to the command line.

```
Running k-Wave line reconstruction...
  grid size: 216 by 778 grid points
  interpolation mode: *nearest
  computation completed in 0.059504s
```

Regardless of the physical alignment of the sensor within the acoustic medium, the reconstruction is always returned as if the sensor was located along the first matrix row (i.e., x = const). The resolution of the reconstruction in the y-direction is defined by the physical location and spacing of the sensor elements, while the resolution in the x-direction is defined by the sampling rate at which the pressure field is recorded (i.e., `dt`). Consequently, the reconstructed initial pressure map typically will have a much finer discretisation in the x- (time) direction. By default, the reconstructed initial pressure distribution is not re-scaled or thresholded in any way. However, a positivity condition can be automatically enforced by setting the optional input parameter `'PosCond'` to `true`.

## The limited view problem

To directly compare the initial pressure distribution with that produced from the reconstruction, it is convenient to interpolate the reconstructed pressure onto a k-space grid with the same dimensions as `source.p0`.

```
% define a second k-space grid using the dimensions of p_xy
[Nx_recon, Ny_recon] = size(p_xy);
kgrid_recon = kWaveGrid(Nx_recon, kgrid.dt * medium.sound_speed, Ny_recon, dy);

% resample p_xy to be the same size as source.p0
p_xy_rs = interp2(kgrid_recon.y, kgrid_recon.x - min(kgrid_recon.x(:)), p_xy, kgrid.y, kgrid.x - min(kgrid.x(:)));
```

The interpolated reconstructed initial pressure distribution (using a positivity condition) with the same plot scaling as the plot of `source.p0` above is shown below. A x = const slice through the center of the larger disc is also shown for comparison. The reconstructed pressure magnitude is decreased due to the limited view problem; the reconstruction is only exact if the data is collected over an infinite line, here a finite length sensor is used.


## Setting the interpolation mode

The FFT reconstruction algorithm relies on the interpolation between a temporal and a spatial domain coordinate with different spacings. This means both the speed and accuracy of the reconstruction are dependent on the method used for this interpolation. This can be controlled via the optional input parameter `'Interp'` which is passed directly to `interp2`. By default, this is set to `'*nearest'` which optimises the interpolation for speed. The accuracy of the interpolation can be improved by setting `'Interp'` to `'*linear'` or `'*cubic'`, and re-running the simulation. This increases the time to compute the reconstruction, however, the noise in the reconstruction is noticeably improved, and the error in the magnitude of the reconstruction is also slightly reduced.


In practice, `'*linear'` interpolation provides a good balance between reconstruction speed and image artefacts.
